# Comment on: Knowledge and Awareness of Tourette’s Syndrome Among Teachers in Eastern Region, Saudi Arabia

**DOI:** 10.5334/tohm.1191

**Published:** 2026-04-02

**Authors:** Sami Ullah Khan, Syed Huzaifa Khan

**Affiliations:** 1 Khyber Medical University, Peshawar, Pakistan

**Keywords:** Awareness assessment, Competence vs performance, Score categorization

## Abstract

**Background:**

We read with interest the article by Alwusaybie et al. examining teachers’ knowledge and awareness of Tourette syndrome in the Eastern Region of Saudi Arabia. The study addresses an important educational and public health issue by assessing teachers’ understanding through a structured questionnaire.

**Main Commentary:**

While the findings provide valuable insight into informational gaps, interpretation requires caution. Questionnaire-based assessments primarily evaluate cognitive knowledge and correspond to the “knows” level of George E. Miller’s pyramid of clinical competence. Such measures may not necessarily reflect applied competence or classroom performance. Therefore, reported knowledge deficits should not be directly equated with inadequate practical preparedness.

Additionally, the use of Bloom’s cutoff criteria to categorize knowledge levels (<60% poor, 60–79% moderate, ≥80% good) may introduce methodological limitations. As highlighted by Douglas G. Altman and Royston, dichotomizing or categorizing continuous variables can result in information loss and potential misclassification, particularly near threshold boundaries. Preserving continuous score interpretation or supplementing categorical reporting with distributional analysis could offer a more nuanced understanding of variability among teachers.

**Conclusion:**

The original study contributes meaningfully to understanding teacher awareness of Tourette syndrome. However, clearer differentiation between knowledge and applied competence, along with cautious interpretation of categorized score thresholds, would strengthen the study’s implications. Future research may benefit from incorporating performance-based assessments and maintaining continuous scoring approaches to better capture real-world educational preparedness.

Dear Editor,

We read with great interest the recent article by Alwusaybie et al [1]. In this study, the authors evaluated knowledge and awareness of Tourrette syndrome among Teachers in Eastern Region, Saudi Arabia. The authors addressed an important social query in which 305 teachers from the Eastern Region were asked 23 questions and based on that they were classified into different types of categories. The study presents promising findings; however, certain aspects of the study need further consideration.

The study primarily evaluates teachers’ knowledge and awareness of Tourette’s syndrome using a questionnaire-based assessment. While such instruments are appropriate for quantifying cognitive understanding, extrapolation of these findings to broader constructs such as preparedness or practical competence warrants careful interpretation. Knowledge-based assessments capture the “knows” level of Miller’s pyramid but may not necessarily reflect applied competence or behavioral performance [2–3]. Consequently, the reported knowledge gaps should be interpreted as indicators of informational deficits rather than definitive measures of real-world classroom preparedness.

Furthermore, the application of Bloom’s cutoff method to categorize knowledge levels may warrant interpretive caution. It categorizes teachers as follows:

1. <60%: poor knowledge of TS
2. 60–79%: moderate knowledge of TS
3. ≥80%: good knowledge of TS

Dichotomizing or categorizing continuous scores at predefined thresholds can result in loss of distributional information and potential misclassification, particularly for values near boundary points [4]. Such categorization may exaggerate perceived knowledge deficits by creating artificial distinctions between participants with marginally different scores. Preserving continuous score interpretation or supplementing categorical reporting with distributional analysis may provide a more nuanced understanding of knowledge variability among teachers.

In summary, the study provides valuable insight into teachers’ knowledge of Tourette’s syndrome within an important educational context. However, careful distinction between cognitive knowledge and applied preparedness, along with cautious interpretation of categorized score thresholds, would further strengthen the implications of the findings. Future investigations may benefit from integrating performance-based assessments or preserving continuous scoring approaches to better capture variability in teachers’ understanding and practical readiness.
